# Rooting for survival: how plants tackle a challenging environment through a diversity of root forms and functions

**DOI:** 10.1093/plphys/kiae586

**Published:** 2024-12-05

**Authors:** Prashanth Ramachandran, Andrea Ramirez, José R Dinneny

**Affiliations:** Department of Biology, Stanford University, Stanford, CA 94305, USA; Howard Hughes Medical Institute, Stanford University, Stanford, CA 94305, USA; Department of Biology, Stanford University, Stanford, CA 94305, USA; Department of Biology, Stanford University, Stanford, CA 94305, USA; Howard Hughes Medical Institute, Stanford University, Stanford, CA 94305, USA

## Abstract

The current climate crisis has global impacts and will affect the physiology of plants across every continent. Ensuring resilience of our agricultural and natural ecosystems to the environmental stresses imposed by climate change will require molecular insight into the adaptations employed by a diverse array of plants. However, most current studies continue to focus on a limited set of model species or crops. Root systems are particularly understudied even though their functions in water and nutrient uptake are likely pivotal for plant stress resilience and sustainable agriculture. In this review, we highlight anatomical adaptations in roots that enable plant survival in different ecological niches. We then present the current state of knowledge for the molecular underpinnings of these adaptations. Finally, we identify areas where future research using a biodiversity approach can fill knowledge gaps necessary for the development of climate-resilient crops of the future.

## Introduction

Biodiversity describes the variety of organisms that inhabit a particular ecological condition or location (Raven and Wackernagel 2020). Beyond the inherent but incalculable value of biodiversity as an inheritance from previous generations, biodiversity also provides practical resources to human society across several fronts (Ehrlich and Ehrlich 1997). Ecosystems with high biodiversity tend to be more stable and resilient to environmental change (Tilman et al. 2014). Biodiversity in our agricultural systems buffers against the effects of disease and abiotic stresses (Keesing et al. 2010). Biodiversity is also evident in the array of plants valued by human cultures worldwide. Despite the evident value of biodiversity, current trends indicate that great losses have already occurred, with even greater losses predicted to lie ahead as climate change takes hold (Corlett 2016). Temperature increases will cause more erratic and severe shifts in weather patterns, challenging our agroecosystems to maintain yields (Gupta et al. 2020). While rising CO_2_ levels can potentially improve crop productivity due to enhanced photosynthesis, these effects are not uniform across species and may have detrimental interactions with other stresses such as drought (Leakey et al. 2009; Gray et al. 2016). Current efforts in crop improvement utilize the knowledge of gene functions gained by studies in molecular genetic model systems such as *Arabidopsis thaliana*. Fueled by major investments from funding agencies, research across the major crops is advancing our understanding of the role that gene regulatory networks play in mediating plasticity to environmental stresses like drought and flooding (Reynoso et al. 2019; Kajala et al. 2021). Nevertheless, these efforts represent only the tip of the iceberg for what is needed to ensure climate resilience across the many economically and culturally important crops worldwide.

The plant root system represents an important frontier in our goal of establishing a more sustainable system of agriculture (Lynch and Brown 2012). Roots are essential for obtaining water and nutrients from the soil and providing mechanical support to the plant body (G Viana et al. 2022). Many of the functions that roots perform can be understood based on the anatomy and cell type–specific functions of root tissues. Roots have also been a highly valuable model system to explore fundamental mechanisms for environmental stress response and their impact on growth and development (Wachsman et al. 2015; Dinneny 2019). Yet the broad relevance of these studies is uncertain as we know little regarding the conservation or transferability of this knowledge across the plant kingdom.

In this review, we aim to invigorate mechanistic research into diverse species by highlighting an array of root adaptations to different, often stressful, ecological conditions that challenge plant physiology. We then highlight current research into the molecular pathways that underlie these adaptations. While this research frequently uses crops and model systems, it nevertheless provides a scaffold of molecular insight into the genes and physiological processes that mediate environmental adaptations of plants. Finally, we provide a future perspective on how the use of a diverse collection of experimentally tractable organisms can advance our understanding of form–function relationships in plant roots and facilitate the development of climate-resilient crops of the future.

### Anatomical basis of root adaptations to stressful environments

Throughout their evolution, vascular plants have established root adaptations to effectively navigate the challenging and dynamic environmental conditions of the rhizosphere (root-associated environment). Whether in dry, aquatic, flooded, nutrient-deficient, or saline habitats, plant species showcase remarkable root-based structures to thrive in diverse ecosystems. Roots become specialized to their environments through changes in root system architecture, morphology, as well as whole root and tissue-specific anatomy. This section aims to explore root adaptations across different organizational levels that have led to the success of plants under diverse environmental challenges.

#### Desert environments

**Figure 1.**
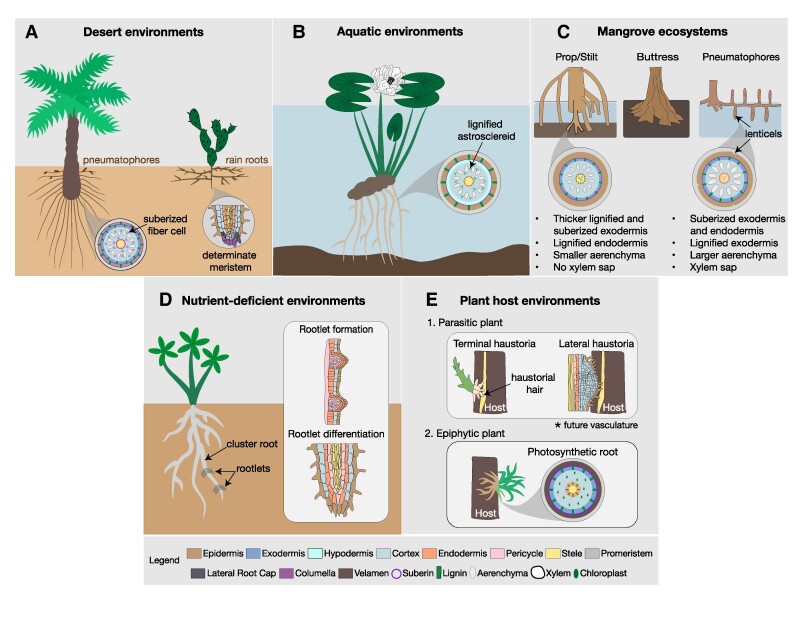
Root system architecture and anatomical responses to different habitats. **A)** Desert environment. A date palm tree on the left and cactus plant on the right. Date palms have secondary root structures called pneumatophores that form near the soil surface. A cross-section diagram of a date palm root showing a suberized epidermis, exodermis, endodermis, and fiber cells. Aerenchyma tissues surrounding the central vasculature (bottom, left). A representation of a determinate meristem that form in some cacti species, with root hairs along the epidermis layer. Figure modified from (Kirschner et al. 2023). **B)** Aquatic environments. A water lily root cross section with a lignified epidermis, endodermis, and astrosclereids. The hypodermis contains suberin. Aerenchyma tissue surrounding the central stele. **C)** Mangrove ecosystem. Common root structures of mangroves species including prop/stilt (left), buttress (middle), and pneumatophores (right). Root cross section diagrams of prop/stilt roots (bottom, left) and pneumatophores (bottom, right), highlighting differences in anatomical structures. **D)** Nutrient-deficient environments. White lupin plant with rootlets stemming from the pericycle of a cluster root. The panel to the right shows the anatomical structures involved in rootlet formation and differentiation. **E)** Plant host environments. 1. Parasitic plants form terminal haustoria (upper left) and lateral haustoria (upper right). An illustration of striga contacting a host root and forming haustorial hairs from the root apical meristem. Haustorial hairs penetrate the host until they reach and make connections with the vasculature of the host. Lateral haustoria differentiation of several root layers. The outermost layer also forms root hairs to secure attachment and invasion of the host. 2. Epiphytic plants attach to a host but do not invade. Some can form specialized photosynthetic roots with a spongy velamen radicum layer, allowing for absorption of moisture and nutrients from the air.

Deserts are classified as having less than 25 cm/y of rainfall (Walker 1996). The rainfall distribution is variable and typically results in high spatial and temporal heterogeneity in environmental water availability (Lynch 1995). Generally, deeper soil layers receive water from winter and spring rainfall, while shallow soil layers rely on summer rainfall or shorter rainfall seasons (Caldwell 1985). Initial studies of desert root systems illustrate that locally adapted plants predominantly develop either deep taproots, shallow lateral root systems, or both to access water across varying depths (Canon 1911). Additionally, some species of succulents like Opuntia and Agave develop “rain roots” (Fig. 1A) that emerge quickly in response to soil moisture and then shed off when the water evaporates (Hunt and Nobel 1987; North et al. 1993; Snyman 2006). These roots are known to have higher hydraulic conductivity and higher potential for water and nutrient uptake (Caldwell 1979; Hunt and Nobel 1987; North et al. 1993; Snyman 2006). As the soil dries it becomes imperative to prevent the backflow of water from roots into the soil. Thus, some rain roots develop aerenchyma, a specialized type of cortex containing large air cavities between cell gaps known as lacunae. Aerenchyma is formed within ground tissues, defining the root cell layers between the outermost epidermal layer and the stele, where the vasculature is housed. Because air is much more resistant to water flow than plant tissue, the lacunae act quickly to lower hydraulic conductivity and limit water loss (North and Nobel 1991, 1992; Dubrovsky et al. 1998). Aside from rain roots, some desert plants like date palms (Arecaceae) develop secondary root structures called pneumatophores to access topsoil water (Seubert 1997). Pneumatophores are agravitropic roots that grow above or below ground (Fig. 1A) near the soil surface (Jost 1887; de Granville 1974; Seubert 1997). They expand the root system surface area, allowing an increase in water uptake during rainfall events (Seubert 1997; Xiao et al. 2019).

Most angiosperms display indeterminate primary root growth involving the continuous self-renewal and maintenance of stem cells throughout the plant's life. However, cacti, in particular those in the Cactoideae subfamily, develop determinate primary roots (Fig. 1A) that only grow for a few days until they have reached full maturation (Dubrovsky 1997; Shishkova et al. 2013). This involves prompt and full exhaustion of their root apical meristems, resulting in rapid differentiation of tissues such as hairs and lateral roots (Dubrovsky 1997; Shishkova et al. 2013). Establishing a broad network of lateral roots early on allows seedlings to take advantage of resources at the soil surface and improve chances of seedling survival. Further exploration of subfamilies within the Cactaceae revealed that most species facing arid to semi-arid environments develop determinate primary roots and meristems, while those facing environments with a moderate supply of moisture are more likely to develop indeterminate primary roots (Dubrovsky 1997; Shishkova et al. 2013).

In addition to variation in root growth and morphology, plants also exhibit specialized cell wall modifications to limit water loss. Cell wall modifications, consisting of complex polymers such as suberin or lignin, are typically present, but not restricted to, the ground tissue layers (cortex and endodermis) in the root. Suberin, a polyester of α, ω-bifunctional fatty acids, and glycerol, is first deposited in a patchy manner in a few endodermal cells but later becomes continuously deposited across all endodermal cells (Bernards 2002). Some species also have a specialized cortex layer termed an exodermis or a hypodermis that is suberized and/or lignified. In Opuntia and Agave species, multiple cell layers show suberized cell walls (epidermis, exodermis, and endodermis) in response to drought (North and Nobel 1991, 1992). The cortex layer in *Phoenix dactylifera* (Date palm) contains suberized fiber bundles that have been proposed to play a role in preventing water loss (Fig. 1A) (Xiao et al. 2019). Fiber cells are also found near xylem vessels in desert species *Zygophyllum album L.F.* and *Nitraria retusa*, where they are thought to function in protecting vascular tissues from embolisms (Abd Elhalim et al. 2016). Lignin, a phenolic polymer, further blocks apoplastic movement of water and ions. Date palms have lignified cell walls in their epidermis, exodermis, and endodermis layers to prevent water loss, regulate ion intake into the vasculature, and provide structural support (Fig. 1A) (Drabble 1904; de Granville 1974; Seubert 1997; Abd Elhalim et al. 2016; Bokor et al. 2019; Xiao et al. 2019).

Desert plants of the Cactaceae, Agavaceae, and Asteraceae family also undergo coordinated anatomical changes that lead to a phenomenon termed root contraction. Although the specific tissues contributing to active contraction may differ between species, root contraction usually results in the shoot being pulled deeper into the soil, which is thought to provide an adaptation to the high temperatures of desert habitats due to the lower relative soil temperature. Furthermore, contractile roots in Agavaceae species have higher hydraulic conductivity, thus aiding in water uptake (North et al. 2008), and may help anchor the plant in rocky soils (Garrett et al. 2010). Root contraction is described as “re-oriented cell growth” via parenchyma cells expanding radially and shortening longitudinally (de Vries 1880). Studies in *Gymnarrhena micranatha* (Asteraceae) showed that endodermis, pericycle, and phloem cells contract to about one-half their length (Zamski et al. 1983). They also showed that the cell walls of these cells become undulated or wavy. Studies in *A. fissaratus* (Cactaceae) showed that xylem conduits may also play a role by forming a lattice-like structure that can compress root length (Garrett et al. 2010).

#### Aquatic environments

In contrast to desert plants, aquatic plants face an abundant water supply. They are typically categorized as emergent, submerged, floating-leaved, or free-floating species (Richards and Sculthorpe 1968; Shin and Choi 1998). Although water availability is not a limitation, aquatic plants face many other challenges, such as nutrient deficiencies, low oxygen availability, and a stable place to root. In this section, we highlight some of the root adaptive strategies of aquatic and wetland plants and how some of these features also facilitate the survival of flooding-adapted species to hypoxic conditions.

Emergent aquatic species are characterized by roots that grow in the substrate and have above-water photosynthetic tissues (Richards and Sculthorpe 1968). The term substrate refers to the type of soil or sediment these plants are rooted in. These species tend to have an extensive root system and are found between open water and more terrestrial habitats like lakes with shallow shorelines (Clarke 2012). The lifecycle of most submerged aquatic plants occurs entirely underwater with less extensive root systems (Clarke 2012). Water lilies are examples of floating-leaved species, which are rooted in sediment but are typically found in shallow waters since their leaves and flowers are above the surface (Del Claro et al. 2009). In freshwater ecosystems, emergent and submerged plants are more likely to obtain their nutrients from sediment due to the high levels of phosphorus and nitrogen typically found in lakes (Barko et al. 1991). Free-floating aquatic plants are not rooted in substrate and may or may not develop roots below the water surface (Del Claro et al. 2009).

A common characteristic for most aquatic plants is the development of a hypodermis or exodermis layer as the outermost ground tissue layer. This specialized cortical layer functions as a barrier for solute uptake. Whether it is termed a hypodermis or exodermis depends on the species. For example, *Alternanthera philoxeroides* roots have a lignified hypodermis and additional cortex layers, which regulate water, oxygen, and solute intake in aquatic environments (Yang et al. 2019). As described by (Seago 2018), the term exodermis is used to refer to a hypodermis containing Casparian strips and suberin lamellae and can be found in many wetland species like *Nymphaea odorata* and *Caltha palustris*. The exodermis—along with astrosclereids, specialized lignified sclerenchyma cells—provide mechanical support in sandy and muddy substrates while protecting aerenchyma (cortical tissues with air spaces) from collapse in *N. odorata* (Fig. 1B) (Seago et al. 2000, 2005). For *C. palustris*, the development of an exodermis in the mature sections of the root may be linked to lowering water levels in the spring, exposing the roots to air (Seago et al. 2000). Similar specialized cell walls are observed in several invasive aquatic species—*Alternanthera philoxeroides* (alligator weed), *Eichhornia crassipes* (water hyacinth), and *Pistia stratiotes* (water lettuce)—but occurring in the cortex and rhizodermis layer, a specialized epidermis, that accumulates and retains ions before the hypodermis forms (Wu et al. 2021).

Aerenchyma is a specialized cortex tissue found in most aquatic plant species and facilitates gas exchange. Expansion of aerenchyma tissue leads to large lacunae, or chambers, which have 3 main modes of origin: schizogeny, lysigeny, and expansigeny (Seago 2018). Schizogeny is the process by which cell–cell separation establishes the lacunae, while lysigeny creates these spaces through programmed cell death of some cortical cells (Jung et al. 2008). Expansigeny is a term that describes the formation and expansion of lacunae from existing small intercellular spaces through cell division and differential cell wall enlargement without further cell separation or death (Seago et al. 2005; Bona et al. 2018). Diversity in the patterns of aerenchyma formation among flowering plants in diverse aquatic and wetland habitats—and their roles in providing aeration, promoting beneficial microbial growth, inhibiting heavy-metal toxicity, and pressurized ventilation—have been extensively described (Seago et al. 2005; Jung et al. 2008; Doležal et al. 2021).

Several root adaptations found in aquatic plants also appear in non-aquatic plants when exposed to flooding, which facilitates acclimation to hypoxia (low oxygen). Rice, for example, is a wetland crop species that induces suberization of its outermost ground tissue layer under waterlogged conditions to reduce radial oxygen loss and prevent the uptake of toxic ions (Watanabe et al. 2013). The suberized exodermal layer in rice also insulates the network of aerenchyma air channels and facilitates oxygen delivery to the root tip to support the growth of this energetically demanding tissue (Yamauchi et al. 2021). Recent studies have found that these barriers and gas spaces are also present in lateral roots and other adventitious roots (Pedersen et al. 2021). Oxygen transport is likely important for flooding tolerance in crops such as wheat and *Z. nicaraguensis* since accessions with a higher proportion of aerenchyma are more likely to be flooding tolerant (Mano and Omori 2013; Herzog et al. 2016).

#### Mangrove ecosystems

Salinity typically harms stress-sensitive plants through mechanisms such as osmotic stress, toxic ion accumulation, nutritional deficiencies, or a combination of these factors (Ashraf 2004; Julkowska and Testerink 2015; van Zelm et al. 2020). Higher levels of sodium ions also cause a reduction in water potential and limit water availability to plant roots (Polle and Chen 2015). Current estimates indicate that one-third of agricultural land is affected by salt stress, highlighting the importance of studying root adaptive mechanisms of salt-tolerant species, or halophytes, to uncover strategies that potentially can be engineered for crop improvement (Cheeseman 2013; Kazachkova et al. 2018).

Mangroves are halophytic species capable of not only surviving high salinity levels but also withstanding flooding, fluctuating tropical temperatures, and heavy metal toxicity (MacFarlane et al. 2007; Agoramoorthy et al. 2008). Mangrove forests dominate intertidal zones along tropical and subtropical shores and are of great ecological importance since they mitigate the effects of tidal waves and floods, thus protecting uplands from more severe damage (Mazda et al. 1997; Peters et al. 1997). Previous studies demonstrate that conferred salt tolerance of halophytes involves the concerted functioning of roots at the cell, tissue, and organ level (Flowers and Colmer 2008; Munns and Tester 2008; Flowers et al. 2010; Shabala and Shabala 2011; Li et al. 2023).

Many mangrove species develop specialized roots to withstand loose sediment and flooding events. Some root types form above the waterline, including prop/stilt, buttress, and pneumatophores (Augustinus 1995; Ong et al. 2004). Prop/stilt roots form as outgrowths from trunks or branches in *R*. *mangle* and *R*. *apiculata* and provide anchorage by creating a wider base and penetrating the soil at multiple points (Fig. 1C) (Srikanth et al. 2016). These roots are submerged at times but usually form at the high points of the trunk to provide anchorage; they can also gather sediment and organic matter carried by water to build soil underneath the mangrove plant (Gill and Tomlinson 1977; Ohira et al. 2013; Srikanth et al. 2016). Buttress roots are large, wide structures (Fig. 1C) and form at the base of trunks to provide further anchorage but also help with the transport of water and nutrients by creating a wide network at the soil/water surface (Crook et al. 1997; Mickovski and Ennos 2003; Méndez-Alonzo et al. 2015). Pneumatophores are found in several mangrove families and are a common root type that develop in flooding conditions. Their gas exchange properties were first studied in the mangrove species *Avicennia nitida* by Scholander et al. (1955). They often develop lenticels, a raised pore, and large aerenchyma/lacunae to facilitate gas exchange in hypoxic conditions (Fig. 1C) (Scholander et al. 1955). Air is transported through pneumatophores to below-ground parts of the root system where oxygen is used for aerobic respiration, with some being released through aerenchyma to the surrounding soil (Srikanth et al. 2016).

The development of apoplastic barriers in mangroves may correlate to the tidal region they occupy (Krauss et al. 2008). For example, pioneer foreshore species like *A. marina* and *Aegiceras corniculatum* are better suited for anaerobic and nutrient-deficient conditions. They have a thin exodermis and large aerenchyma allowing them to supply oxygen to submerged roots while creating aerobic conditions in aerial roots (Fig. 1C) (Armstrong and Wright 1975; Colmer 2003; Pi et al. 2009; Cheng et al. 2014). Imaging and X-ray microanalysis shows that increases in salinity levels in *A. marina* and *A. officianalis* exhibit increased suberin deposition in the exodermis and endodermis, which helps reduce Na^+^ loading into the xylem (Krishnamurthy et al. 2014; Cheng et al. 2020). *Rhizophora* species such as *Kandelia obovata* and *Rhizophora stylosa* have a thicker lignified and suberized exodermis layer allowing them to cope with salt and heavy metal toxicity and smaller aerenchyma since they face less inundation (Fig. 1C) (Liu et al. 2009; Cheng et al. 2010, 2014). Deeper tissue layers of mangrove roots exhibit additional anatomical modifications. For example, *R. mangle* and *A. marina* roots contain mucilage in their xylem to block water movement as it is transported to the shoots and prevent salt accumulation (Fig. 1C) (Zimmermann et al. 1994; Zimmermann et al. 2002; Cheng et al. 2020). The viscous xylem sap is made up of mucopolysaccharides, which slow down the rate of transpiration and bind salt ions limiting build-up in the surrounding rhizosphere (Ball 1988; Zimmermann et al. 1994; Zimmermann et al. 2002).

#### Nutrient-deficient environments

Mineral nutrients are crucial for plant growth and yield. Cultivating productive crops requires sufficient nutrient availability from the soil (Lawlor 2004). However, nutrient-deficient environments are found in various regions across the globe and are often influenced by specific climatic and geological conditions. For example, tropical rainforests have nutrient-deficient soils due to heavy rainfall causing the leaching of nutrients (Davies 1997). Rainforests are also subject to extreme weathering leading to acidification of soils and release of micronutrients toxic to plants (Sanchez and Logan 2015). Desert soil is also nutrient-poor due to low rainfall decreasing nutrient mobility along with low organic matter and nitrogen (Archibold 2012; McHugh et al. 2017). Moreover, cold habitats such as tundra experience low fertility due to limited plant production hindering decomposition and microbial nitrification, thereby reducing nitrogen cycling (Haag 1974).

Cluster roots are a common adaptation in soils of low fertility. They were first discovered in the *Proteaceae* family, but other plant families like the *Betulaceae*, *Casuarinaceae*, *Cucurbitaceae*, *Cyperaceae*, *Eleagnaceae*, *Leguminosae*, *Moraceae*, *Myricaceae*, and *Restionaceae* families, which are all adapted to nutrient-deficient landscapes, also develop these or similar root types (Purnell 1960; Skene 2000). Cluster, or proteoid, roots are described as having “bottlebrush-like” rootlets that arise from the pericycle and have determinate growth with dense root hairs at the meristem (Shane and Lambers 2005) (Fig. 1D). They are usually induced by a shortage of phosphorus but can also be induced by iron (Fe) deficiency in some species (Shane and Lambers 2005). Additionally, they can be found in nutrient-rich pockets where lateral roots are initiated and help maintain a high level of nutrient mobilization (Neumann and Martinoia 2002). Studies in *Lupinus albus* have also shown that during cluster root development, large amounts of root exudates like carboxylates, protons, phenolics, and acid phosphatases are released into the rhizosphere, which may contribute to more efficient use of available phosphorus and potentially recruit beneficial microbial communities (Gardner et al. 1981; Lambers et al. 2006).

Anatomical plasticity can also be observed under varying nutrient levels. Under low phosphorus and low iron conditions, epidermal cell files generate longer and denser root hairs, aiding in the uptake of immobile nutrients (Schmidt Tittel and Schikora 2000; López-Bucio et al. 2003; Cruz-Ramrez et al. 2009; López-Bucio et al. 2018). This is explained by an increase in the number of epidermal cells that differentiate into trichoblasts, or root hair precursor cells (Bates and Lynch 1996; Ma et al. 2001; López-Bucio et al. 2003). Although this has mostly been studied in *A. thaliana*, studies in tomato, spinach, and rape show similar effects under low P and low N (Foehse and Jungk 1983; Jungk 2001). Aerenchyma has also been shown to reduce root metabolic costs and increase soil exploration in maize and the common bean (Lynch et al. 2005; Saengwilai et al. 2014; Zhan and Lynch 2015). Aerenchyma formation is beneficial under multiple nutrient deficiencies, including phosphorus, nitrogen, and sulfur (Lynch and Brown 2008). Hydrophobic barriers in endodermal cell files have also been shown to respond to varying nutrient concentrations. In *A. thaliana*, suberin deposition is increased under potassium and sulfur deficiency and decreased under iron, zinc, and magnesium deficiency (Andersen et al. 2015; Barberon et al. 2016).

#### Plant host environments

Plants themselves can serve as hosts for other plants through parasitism or epiphytism. Parasitic plants derive some or all their nutritional requirements from other living plants. They often have significant ecological and economic impacts since they can damage or kill their host plant. Parasitic weeds alone are estimated to cause losses of over $2 billion annually in crop yields worldwide (DiTomaso 2000). A deeper understanding of parasitic root systems can offer insights into their ecological effects on community diversity and to potentially guide strategies to mitigate the adverse impacts they have on ecosystems (Ameloot et al. 2005).

Root parasitic plants begin their lifecycle when they sense haustorium-inducing factors from a compatible host, which results in the formation of a pre-haustorium or a host-penetrative root (Cui et al. 2018). Subsequent development of intrusive cells of the prehaustorium eventually leads to haustorium formation, which acts as a conduit between the vasculature of the parasite and host (Losner-Goshen 1998). There are 2 types of haustoria: terminal and lateral haustorium. Terminal haustoria arises from the differentiation of the root apical meristem in parasitic plants (Yoshida et al. 2016). As they develop, cell division in the meristem slows, and the differentiation of epidermal cells leads to the formation of haustorial hairs, which penetrate the host's root tissues (Fig. 1E-1) (Hood et al. 1998; Yoshida et al. 2016; Xiao et al. 2022). Eventually, these structures reach the endodermis, where the outermost cells elongate and divide to form a palisade layer and xylem elements differentiate to establish vascular connections to the host (Hood et al. 1998). Lateral haustoria usually develops after terminal haustoria, originating from secondary adventitious roots to enhance nutrient uptake from the host (Cai et al. 1993). Their formation triggers cell division in the pericycle, endodermis, cortex, and epidermis layers (Fig. 1E-1) (Kirschner et al. 2023). Like in terminal haustoria, the outermost layer divides, and root hairs form to secure attachment to the host (Musselman and Dickison 1975). A hyaline body then forms at the center of lateral haustoria, containing parenchyma cells with high metabolic activity that serve as a sink tissue for host metabolites (Visser et al. 1984; Yoshida et al. 2019).

**Figure 2.**
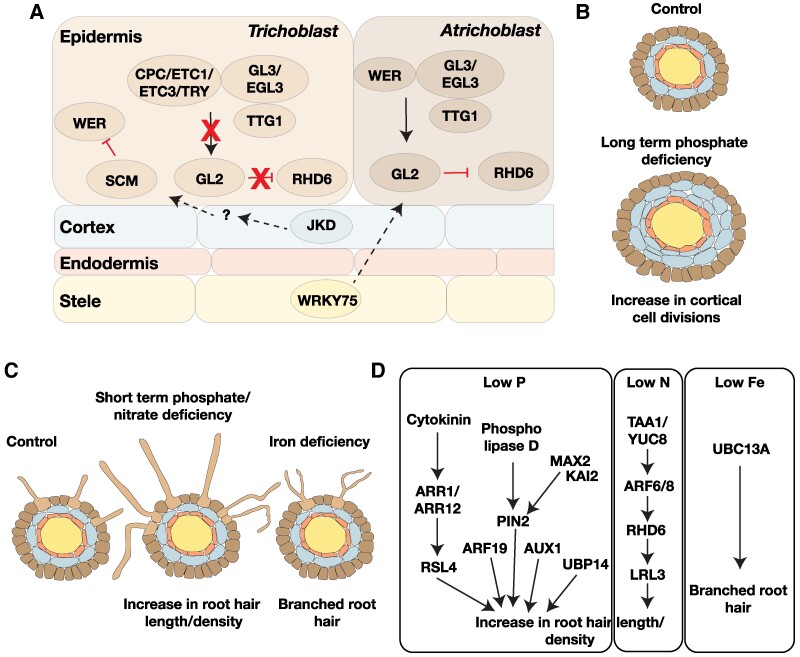
Genetic pathways controlling root hair plasticity. **A)** Genes involved in patterning of the root epidermis identified through studies in *A. thaliana*. In atrichoblast cells, a protein complex consisting of WER, TTG1, and GL3/EGL3 activates the transcription factor *GL2* to repress root hair formation. The absence of WER in trichoblast cells results in a protein complex that is unable to activate *GL2* to allow root hair development. The patterning of the root epidermis is also influenced by non-cell autonomous functions of *JKD* (from the cortex) and *WRKY75* (from the stele). **B)***A. thaliana* roots exposed to extended periods of phosphate deficient conditions show increased cortical cell divisions. This in turn influences the number of trichoblast cells in the epidermal cell layer, resulting in an increase in root hair number in phosphate-deprived conditions (Janes et al. 2018). **C)** Adaptation to short-term phosphate and nitrate deficiency results in increased root hair length and density, while iron deficiency causes branched root hairs (Müller and Schmidt 2004). These changes increase the root surface area available for nutrient absorption. **D)** Auxin and cytokinin signaling pathways influence root hair development under phosphate- and nitrate-deficient conditions. Auxin regulates root hair development through ARF19 in low-phosphate conditions, while changes under low-nitrate conditions depend on ARF6/8. The ubiquitin ligase UBIQUITIN-CONJUGATING ENZYME controls branching of root hairs observed under iron-deficient conditions.

Although epiphytic plants also attach to other plants, they do not depend on these plants for nutrients or water but rather for physical support. Epiphytism has independently evolved in several nonrelated plant taxa, but most are encompassed in 23 families, with a majority being in the Orchidaceae (Kress 1986). Orchids produce aerial roots to anchor themselves to trees. These specialized adventitious roots do not have a root cap or root hairs but instead have 1 or more spongy layers of epidermis, called velamen radicum, which facilitates moisture and nutrient absorption from the air (Fig. 1E-2) (Shekhar et al. 2019). The velaman functions as temporary water storage until it can be transported across the exodermis to the vascular system. Roots of the orchid *Phalaenopsis amabilis* contain a suberized and lignified exodermis preventing leakage of water from the cortex (Fig. 1E-2) (Haberlandt 1904; Fahn 1990; Evert and Eichhorn 2006).

Certain orchid species have chloroplast-containing aerial roots, which suggests they can photosynthesize. Previous findings suggest, however, that the process involves internal refixation of respiratory CO_2_ rather than a net photosynthetic process (Aschan and Pfanz 2003). Recently, (Brunello et al. 2024) demonstrated that the aerial roots of orchids (Phalaenopsis) experience hypoxic conditions in darkness due to their substantial bulk, which impedes efficient oxygen diffusion. Under light conditions, photosynthetic pigments within the roots generate oxygen, thereby mitigating hypoxia (Brunello et al. 2024).

Some epiphytes, like aroid vines, begin their life cycle as terrestrial plants and then transition toward the canopy. This transition requires the formation of specialized aerial roots that can be further classified into anchor and feeder roots. Upon ascendance, anchor roots secure the vine to the host trunk, while feeder roots descend and form a connection with the soil (Croat 1988). Both root types grow larger as they mature, with a significant expansion in the diameter of their existing xylem vessels, which enhances hydraulic conductivity (Filartiga et al. 2018).

### Molecular mechanisms controlling plant root adaptations

The diverse range of morphological adaptations (Fig. 1) evolved to handle distinct environmental challenges provides us with a catalog of potential traits that can be engineered into crop species to meet the increasing demands for energy and nutrition. However, the successful transfer of these adaptive traits will require a comprehensive understanding of the molecular mechanisms controlling the development of these morphologies. In the following sections, we will describe the genetic pathways that have been identified, through studies mostly carried out on model species, to control the formation of solute, oxygen, and water transport barriers and morphogenesis of specialized roots for nutrient and water uptake in limiting environments.

#### Plasticity in root hair development under nutrient limitation

Changes in the number and length of root hairs directly influence the available surface area for absorption of water and nutrients. The characterization of regulatory pathways that influence root hair patterning and differentiation in *Arabidopsis thaliana* provides us with clues on how plasticity in root hair development can be brought about in diverse plant species (Fig. 2A). The root epidermis of most plant species consists of trichoblast (cells that are capable of producing root hairs) and atrichoblast (cells that do not produce root hairs) cells; however, the spatial distribution of the hair and non-hair cells within the epidermis can vary between species (Pemberton et al. 2001; Salazar-Henao et al. 2016). In *A. thaliana*, the root hair fate is repressed in the atrichoblast cells by the expression of a homeodomain transcription factor, *GLABRA2* (*GL2*) (Masucci et al. 1996). The expression of *GL2* is activated by a protein complex consisting of WEREWOLF (WER), GL3 or ENHANCER OF GL3 (EGL3), and TRANSPARENT TESTA GLABRA1 (TTG1) (Schiefelbein et al. 2009).

In the trichoblast cells, WER levels are reduced by a signaling pathway that originates from the underlying cortical cell layer. This reduction in the abundance of WER allows the formation of a protein complex, consisting of CAPRICE (CPC)/ENHANCER OF TRY AND CPC/ETC3/TRYPTICHON, GCL3/EGL3, and TTG1, that cannot activate *GL2* (Schiefelbein et al. 2009). Intercellular communication plays an important role in cell fate specification of the epidermal lineage as well. JACKDAW (JKD), a transcription factor expressed in the cortex, activates the expression of a yet unknown signal that determines hair cell fate by restricting GL2 expression (Hassan et al. 2010). The patterning of the epidermal tissue is stabilized further by the movement of CPC from the atrichoblast to trichoblast and the movement of GCL3/EGL3 in the opposite direction (Savage et al. 2008). In addition to this, a vasculature-expressed transcription factor, WRKY75, moves from the inner tissue layers to the epidermis to regulate epidermal cell fate (Rishmawi et al. 2014). Even though the positioning of the trichoblasts with respect to the cortex is similar across many plant families (Clowes 2000), the non-cell autonomous regulation of epidermal patterning by the underlying cortex and stele cells has only been shown in *A. thaliana*. It is currently unknown if these same regulators of cortical origin also influence root hair fate in other species.

The initiation of root hairs, in the cells specified as trichoblasts, is regulated by the transcription factors ROOT HAIR DEFECTIVE6 (RHD6) and RHD6-LIKE1 (RSL1). In non-hair cells, GL2 binds to the promoters of *RHD6* and *RSL1* and represses root hair development (Lin et al. 2015). Polar cell growth, which eventually determines the length of root hairs, is dependent on the control of root hair–specific cell wall expansin genes by the bHLH transcription factor, RSL4 (Vijayakumar et al. 2016).

Studies in *A. thaliana* have shown that several factors such as salinity, drought, temperature, and nutrient availability can regulate root hair development. Here, we will focus on the gene regulatory networks involved in the plasticity of root hair formation under nutrient-limiting conditions, specifically a lack of inorganic phosphorus, nitrogen, and iron (Fig. 2).

*A. thaliana* roots grown under phosphate-limiting conditions for extended periods of time increased the number of cortical cell files in the primary root from 8 to 12, resulting in a higher number of epidermal cells in the hair cell position, thereby increasing root hair density (Janes et al. 2018) (Fig. 2B). Short-term growth in low phosphate (P) conditions has also been shown to increase root hair density independent of an increase in cortex cell number (Fig. 2C). In this case, the change in root hair density was driven by the diffusion of cytokinin that is biosynthesized in the vasculature to the epidermis (Wendrich et al. 2020). Further, cytokinin signaling regulators, B-type response regulators, ARABIDOPSIS RESPONSE REGULATOR1 (ARR1) and ARR12, bind to the promoter of *RSL4* and activate *RSL4* expression to increase root hair growth (Takatsuka et al. 2023; Takatsuka et al. 2024). In addition to cytokinin, auxin biosynthesis, auxin influx in epidermal cells, and auxin-dependent transcription factors, AUXIN RESPONSE FACTOR19 (ARF19), RSL2 and RSL4 have been implicated in the increase of root hair density under P-limiting conditions (Bhosale et al. 2018; Giri et al. 2018).

The effect of auxin influx carrier, AUXIN RESISTANT (AUX1), on root hair elongation has been shown in rice plants grown under low P environments, suggesting that this regulation is likely widespread across several plant species (Bhosale et al. 2018; Giri et al. 2018). A comparison of RSL4 protein levels under low phosphate and replete phosphate conditions identified RSL4 protein lifetime as a contributor of root hair length. During low phosphate conditions, increases in RSL4 synthesis caused a longer lifetime and hence an increase in root hair length (Datta et al. 2015). MORE AXILLARY GROWTH2 (MAX2)-mediated strigolactone signaling in the endodermis regulates an increase in root hair density non–cell autonomously under low P conditions in an auxin receptor TRANSPORT INHIBITOR RESPONSE 1–dependent manner (Mayzlish-Gati et al. 2012; Madmon et al. 2016). MAX2 has also been found to function with KARRIKIN INSENSITIVE2 under low P conditions to increase auxin transport (PIN2 and AUX1)-dependent root hair elongation (Villaécija-Aguilar et al. 2022). PIN2 protein levels in the epidermis are elevated in low P conditions by the suppression of PIN2 degradation. This suppression of PIN2 degradation is dependent on phospholipase D–derived phosphatidic acid signaling, suggesting membrane-lipid remodeling during low P conditions can influence root hair development (Lin et al. 2020).

Mutations in a ubiquitin protease, UBP14, have been shown to prevent the elongation of root hairs under low phosphate conditions, suggesting that ubiquitin-mediated degradation of root hair–regulating proteins is key to low phosphate response (Li et al. 2010). The identification of UBP14 targets will be key to understanding how regulation at the protein level influences developmental plasticity of root hairs.

It would be interesting to test if species that have adapted to survive in phosphate-deficient soil have a higher number of cortical cells and hence an increased root hair density or if this is due to changes in cytokinin, strigolactone, or auxin signaling. Local auxin biosynthesis in the root through TRYPTOPHAN AMINOTRANSFERASE OF ARABIDOPSIS 1/YUCCA8 and transport into the epidermis through PIN2 and AUX1 also plays an important role in the stimulation of root hair elongation under nitrogen deficiency. The epidermal auxin signaling mediated by ARF6/8 promotes RHD6 accumulation to activate its downstream target, *LOTUS JAPONICA ROOT HAIRLESS-LIKE 3* to modulate root hair elongation (Jia et al. 2023). Thus low N and P act through auxin to enhance root hair elongation but through different ARFs (Fig. 2D).

In contrast to the increase in root hair density and elongation observed in response to N and P starvation, *A. thaliana* plants grown on iron-deficient conditions form branched root hairs to increase the surface area similar to those observed in *auxin resistant 1* (*axr1*), *axr2*, and *aux1* mutants (Grierson and Schiefelbein 2002; Müller and Schmidt 2004). The formation of branched root hairs is dependent on UBIQUITIN-CONJUGATING ENZYME, indicating protein turnover of its targets could potentially regulate branched root hair formation (Li and Schmidt 2010) (Fig. 2, C and D).

#### Mechanisms for ground tissue formation and functional diversification

The water and nutrients that are taken up by the epidermal cell layer are transported through the root cortex to the inner vascular tissues. The path length these molecules traverse depends on the number of cortical cell layers; however, how variation in the number of cortex cell files influences nutrient uptake and retention has not been extensively studied. The composition of the ground tissues in plant roots, which include cell types such as cortex, aerenchyma, endodermis, and exodermis, varies depending on the plant species and the environment they are exposed to.

#### Asymmetric cell divisions and cell–cell communication establish ground tissues

**Figure 3.**
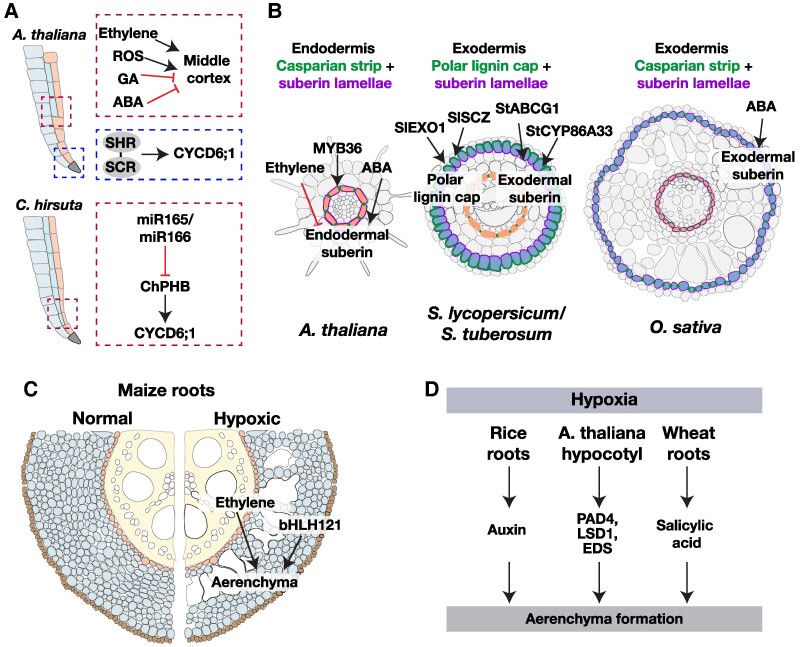
Pathways involved in patterning and differentiation of the ground tissue. **A)** In *A. thaliana* primary roots, a target of the SHR-SCR protein complex, *CYCD6;1*, controls division of the cortex-endodermal initial cell, resulting in the 2 cell files of the ground tissue (cortex and endodermis). In older roots, periclinal divisions in the endodermal cell file form an extra ground tissue layer usually referred to as the middle cortex. The formation of the middle cortex is regulated by the hormones ethylene, gibberellic acid (GA), and abscisic acid (ABA) as well as reactive oxygen species (ROS). In another *Brassicaceae* species, *C. hirsuta*, the middle cortex development occurs earlier in development and is regulated by the transcription factor PHB. The expression of miR165/166 was found to be lower in the endodermis of this species compared with *A. thaliana*, allowing the expansion in expression domain of its target, PHB. **B)** A differentiated endodermis in *A. thaliana* roots consists of 2 barriers for solute transport, the lignin-rich Casparian strip and suberin lamellae (left). *S. lycopersium* and *S. tuberosum* roots also contain a similar endodermal layer (middle). In addition to this, these species also contain an outer exodermal layer that displays a characteristic polar lignin cap and suberin lamellae. In *O. sativa*, the exodermis contains Casparian strip and suberin lamellae similar to the endodermis (right). **C)** Ethylene levels increase in maize roots experiencing hypoxic conditions caused by water-logging. This elevated ethylene triggers cell death leading to the formation of aerenchyma. The transcription factor, bHLH121, promotes the development of aerenchyma in maize roots. **D)** Hypoxic conditions also trigger aerenchyma formation in the rice and wheat roots as well as in *A. thaliana* hypocotyls, with auxin and salicylic acid playing key roles in this process.

The knowledge about genes regulating ground tissue patterning is largely from studies in the model plant *A. thaliana*, which, in early stages of development, features a single cortex and endodermal cell file. The 2 cell files originate from a single initial stem cell, the cortex-endodermis initial. The asymmetric division of the cortex-endodermal daughter (CED) is dependent on 2 transcription factors: SHORTROOT (SHR) and SCARECROW (SCR) (Di Laurenzio et al. 1996; Helariutta et al. 2000; Nakajima et al. 2001) (Fig. 3A). While the *SHR* mRNA is transcribed in the stele, the SHR protein is mobile and moves from the inner cell layers to the cortex-endodermis initial, where division is triggered by the formation of the SHR–SCR protein complex and subsequent activation of a cyclin gene, *CYCLIN D6;1* (*CYCD6;1*) (Sozzani et al. 2010). The sequestration of SHR by SCR restricts its movement further outward and ensures a single division of the CED in *A. thaliana* (Gallagher et al. 2004). The robust patterning of the ground tissue is facilitated by the reinforcement of the SHR-SCR-CYCD6;1 regulatory module through the control of SCR activity by RETINOBLASTOMA RELATED (RBR), activation of *CYCD6;1* by auxin and the control of RBR phosphorylation by CYCD6;1 (Cruz-Ramírez et al. 2012; Winter et al. 2024).

It has been proposed that another stele-derived signal acts in concert with SHR to specify the endodermis. Ectopic expression of *SHR* or the expression of rice and *B. distachyon SHR* under the *AtSHR* promoter in *A. thaliana* results in multiple cell files with a single endodermis and multiple cortex layers (Wu et al. 2014). The formation of multiple cortex cell files has been attributed to increased mobility of BdSHR and OsSHR1 proteins over several cell layers. In *Cardamine hirsuta*, a close Brassicaceae relative of *A. thaliana*, the formation of 2 cortical layers was found to be dependent on the reduction in the levels of active microRNAs, miR165/166, from the CED, causing an expansion in the expression domain of the miRNA target, *PHABULOSA* (*PHB*) (Di Ruocco et al. 2018) (Fig. 3A). Interestingly, the expression of *ChSHR* in *A. thaliana* also caused an increase in the number of cortical cell files, suggesting that the SHR-SCR and miR165/166-PHB pathways represent 2 independent mechanisms to achieve similar phenotypic outcomes on cortical tissue patterning. While the vascular and endodermal expression domains of *SHR* and *SCR* orthologs are largely conserved across several species, scRNAseq analysis of maize roots showed that both *SHR* and *SCR* orthologsf were expressed in the endodermis. Similar expansion of *SHR* mRNA expression domain into the endodermis and cortex has also been observed in date palm (*Phoenix dactylifera*) (Xiao et al. 2019). However, the *Zmshr1/2* double mutants showed a reduction in the number of cortical cell files, suggesting a functional conservation of these genes despite their expression divergence (Ortiz-Ramírez et al. 2021).

Depending on the developmental stage, *A. thaliana* roots can have either 1 or 2 cortex cell files. The development of the extra cortex cell file that is present in older roots, referred to as middle cortex, is regulated by the signaling of hormones gibberellic acid, abscisic acid, and ethylene, as well as ROS (Cui et al. 2014; Cui 2016; Li et al. 2020; Oh et al. 2023) (Fig. 3A). In addition to these regulatory components, the hormone auxin has also been found to control divisions in the cortex layer in *A. thaliana*. An increase in auxin response, specifically in the cortex layer, by the expression of a truncated AtARF5 protein resulted in both periclinal and anticlinal divisions in the cortex layer (Kim et al. 2022). It is unclear how these pathways might contribute to differences in the number of cortex cell files in different species or whether the 2 cortex cell layers have distinguishable physiological roles.

#### Multiple mechanisms for aerenchyma formation

Hypoxic conditions have been shown to enhance ethylene biosynthesis in several plant species (Voesenek and Sasidharan 2013). The role of ethylene in lysigenous aerenchyma formation was first identified in maize roots, where ethylene-dependent cell death activation was critical for aerenchyma formation (Fig. 3C). The conservation of ethylene's role in this process is illustrated by the enhancement of aerenchyma formation in wheat seminal roots under oxygen-deficient conditions (Yamauchi et al. 2014). The identification of other downstream components in this pathway was facilitated by transcriptomic analyses on laser microdissected cortical cells of ethylene-treated (Takahashi et al. 2015) and waterlogged (Rajhi et al. 2011) maize roots. Functional characterization of promising candidates from these transcriptome studies will help identify regulators of specific steps in aerenchyma development. The importance of ethylene-dependent aerenchyma in flooding tolerance was studied by comparison of species of differing tolerance to waterlogging. The tolerant *Dendranthema zawadskii* plants produced larger amounts of ethylene and exhibited faster lysigenous aerenchyma formation compared with the less tolerant species *Dendranthema nankingense* (Yin et al. 2013). A similar comparison between tolerant and sensitive sweet potato varieties showed that in the tolerant variety, ethylene levels, aerenchyma area, and aerenchyma number were significantly higher when exposed to hypoxic stress (Pan et al. 2021).

In addition to ethylene, other signaling pathways can also contribute to aerenchyma formation, depending on the species (Fig. 3D). Studies on the development of lysigenous aerenchyma in rice revealed a role for auxin in this process. Inhibition of polar auxin transport and the expression of the dominant-negative version of the auxin signaling repressor, IAA13, caused a reduction in constitutive aerenchyma formation (Yamauchi et al. 2019) (Fig. 3D). While *A. thaliana* does not form aerenchyma in the root, the Ws-0 ecotype forms aerenchyma in the hypocotyl when exposed to hypoxic conditions, which is dependent on the transcription factor LESION SIMULATING DISEASE 1 and the immune response regulators PHYTOALEXIN DEFICIENT4 (PAD4) and ENHANCED DISEASE SUSCEPTIBILITY1 (Mühlenbock et al. 2007) (Fig. 3D). It would be interesting to investigate whether the orthologs of these regulators control cortical aerenchyma formation in monocots. PAD4 and EDS play key roles in salicylic acid signaling during pathogen infection and salicylic acid enhances aerenchyma formation in both control and waterlogged wheat plants, suggesting that similar components may regulate this process in wheat roots and the *A. thaliana* hypocotyl (Koramutla et al. 2022). Schneider et al. investigated the genetic basis of variation in cortical aerenchyma using 436 diverse maize lines and identified the bHLH transcription factor bHLH121 as a positive regulator of aerenchyma formation (Schneider et al. 2023) (Fig. 3C). A comparison of the downstream regulatory network between species that either lack aerenchyma or species that develop aerenchyma can provide clues to the evolution of this anatomical trait.

While schizogenous aerenchyma formation has been observed in several species (Ishizaki et al. 2013; Ishizaki 2015), the molecular regulation of their formation is less understood. In a *Marchantia polymorpha* T-DNA mutant screen, the plasma membrane-localized E3 ligase MpNOPPERABO1 was identified to play a role in the formation of schizogenous intercellular spaces (Ishizaki et al. 2013).

#### Defining the unique cell wall features and associated functions of the endodermis

The first step in the differentiation of the endodermis involves the formation of a hydrophobic, apoplastic diffusion barrier composed mainly of lignin (Fig. 3B). The CASPARIAN STRIP DOMAIN PROTEIN (CASP) 1–5 recruit peroxidases and laccases to the primary cell wall of the endodermis and along with 2 stele-derived peptides, CASPARIAN STRIP INTEGRITY FACTORS (CIF1 and CIF2), determine the site of lignin deposition. Nearly all of the genes essential for Casparian strip deposition act downstream of SHR, and ectopic expression of SHR and CIF peptides can establish barrier formation in non-endodermal cells (Drapek et al. 2018; Li et al. 2018). While the final stages of endodermal differentiation are marked by suberization of all endodermal cells, some endodermal cells, referred to as passage cells, remain unsuberized and may serve as the gateway for entry and exit of water and nutrients (Andersen et al. 2018) (Fig. 3B). In Casparian strip–defective *A. thaliana* mutants, endodermal suberization is enhanced, suggesting that the regulation of these 2 aspects of endodermal development are tightly interconnected. This interconnection is mediated, in part, through the MYB domain-containing transcription factor, MYB36, that occupies a central position, downstream of SHR/SCR, in the endodermal regulatory network (Liberman et al. 2015; Drapek et al. 2018). Apart from the key transcriptional regulators, signaling of the hormone, abscisic acid (ABA), is important for normal endodermal suberization (Fig. 3B).

The importance of endodermal barriers become more striking when plants are challenged with adverse environmental programs. In maize primary roots exposed to elevated levels of sodium chloride (NaCl), the Casparian strip becomes thicker compared with roots grown under control conditions, suggesting that this might play an adaptive role in the exclusion of NaCl from entering the stele (Karahara et al. 2004; Wang et al. 2022). In support of this notion, a study using maize inbred lines identified *ZmENHANCED SUBERIN LIKE* (*ZmESBL*) as a genetic regulator of Casparian strip thickness and transpiration-dependent sensitivity to salt stress (Wang et al. 2022). The *ZmESBL* mutant roots had similar growth rates and suberin deposition compared with wild-type plants but were defective in CS development and did not display the NaCl-induced increase in CS thickness. This defect likely caused the accumulation of Na^+^ in tissues when mutant plants were exposed to elevated NaCl levels under a transpiring environment. The mutants of *A. thaliana* orthologs of *ESBL* also displayed similar phenotypes and sensitivity to salt stress, exemplifying the importance of Casparian strip for salt tolerance across plant lineages (Wang et al. 2022).

The suberization of the endodermis is also environmentally responsive, with the 2 hormones ABA and ethylene being the main players that regulate suberin biosynthesis or degradation, respectively (Barberon et al. 2016). The ABA-dependent increase in suberization is observed in response to elevated NaCl levels as well as a decrease in potassium and sulfur. In contrast, ethylene-mediated suberin disappearance occurs under iron-, manganese-, and zinc-deficient conditions. The developmental flexibility of these diffusion barriers plays a key role in maintaining nutrient and ion homeostasis and would likely be key for the adaptation of a species to new environments. The comparison of seminal root osmotic stress response in wild (*Hordeum vulgare spp. spontaneum*) and cultivated barley (*Hordeum vulgare spp. vulgare*) showed that while endodermal suberization is enhanced in the root elongation zone of cultivated barley varieties, this enhancement is absent in wild barley (Kreszies et al. 2020), suggesting that domestication of crop species might have led to changes in endodermal responses to the environment.

#### Developmental mechanisms for exodermal differentiation

Single-cell RNA sequencing of *Oryza sativa* (rice) seedling roots identified a cluster of cells that had exodermal identity. Integration of the rice and *A. thaliana* scRNAseq datasets showed that there was a transcriptional similarity between the rice exodermis and the *A. thaliana* endodermis, suggesting a functional conservation between the 2 tissues (Zhang et al. 2021). ABA, as in *A. thaliana* endodermis, was required for exodermal suberin lamellae formation in water-logged rice crown roots and induced exodermal suberization when exogenously supplied to aerated roots (Shiono et al. 2022) (Fig. 3B). In *S. lycopersicum* (tomato) roots, suberin was present only in the exodermis, and the exodermal suberization was enhanced by ABA causing the formation of suberized exodermis closer to the root tip (Fig. 3B). However, *S. pennellii*, a drought-tolerant wild tomato species, exhibited constitutively high suberin levels, which were not altered in response to ABA (Cantó-Pastor et al. 2024).

The transcriptional network downstream of ABA that is responsible for an increase in suberization was conserved between *A. thaliana* (in endodermis) and *S. lycopersicum* (in exodermis), suggesting that the genetic wiring responsible for suberin plasticity in the 2 spatially distinct cell types is similar. In contrast to this, lignin deposition in the *A. thaliana* endodermis and tomato exodermis occurs through distinct pathways. Lignin in the tomato exodermis is deposited as a polarized cap on the outward-facing side of the exodermis and is not regulated by the Casparian strip lignin regulators *SlSHR*, *SlMYB36*, *SlCASP1*, and *SlCASP2* (Manzano et al. 2022). The restriction of polar lignin cap formation to the exodermis was dependent on the transcription factors SlEXO1 (Solyc09g011120) and SlSCZ (Solyc04g078770). Mutations in these genes resulted in an ectopic lignin cap forming in the inner cortex cell or caused nonpolar lignin deposition in the exodermis (Fig. 3B). Studies in another polarized exodermal lignin cap containing Solanaceae species, *Solanum tuberosum* (potato) have shown that exodermal suberization occurs earlier than endodermal suberization (Fig. 3B). The downregulation of the ABCG transporter, *StABCG1*, and suberin biosynthesis gene, *StCYP86A33*, reduces the suberin levels in the potato exodermis and altered the distribution of ions in the root and shoot (Serra et al. 2009; Landgraf et al. 2014; Company-Arumí et al. 2023). Considering salt-adapted mangrove species such as *Avicennia officinalis* enhance exodermis suberization in response to salt, the identification of these genetic regulators provides avenues for the investigation of the functional role of these barriers in adaptation to different environmental conditions (Krishnamurthy et al. 2014).

#### Mechanisms for proteoid root differentiation

The molecular regulators of proteoid root development have primarily been identified through genetic studies in *Lupinus albus*. To aid the characterization of developmental stage-dependent regulators, Thanh et al. and Gallardo et al. have characterized the ontogeny of cluster roots and divided cluster root development into discrete stages (Gallardo et al. 2020; Le Thanh et al. 2021). Similar to the lateral roots of *A. thaliana*, the initial steps of rootlet formation begin with divisions of the pericycle in proteoid roots. Transcriptional reporter analysis suggested that cell type markers such as *LaSCR*, *LaWOODENLEG* (*LaWOL*), *LaPEP*, and *LaEXPANSIN7* (*LaEXP7*) retained similar cell type specificity as their orthologs in *A. thaliana*. RNAi-mediated downregulation of *LaSCR1* and *LaSCR2* caused a reduction in cluster root number, and reporter analysis showed that both paralogs retained their endodermal expression specificity (Sbabou et al. 2010). However, *LaWOX5.2-like*, whose ortholog is expressed specifically in the quiescent center cells of *A. thaliana* roots, showed an expanded vascular expression domain during later stages of rootlet development (Le Thanh et al. 2021). Future characterization of *LaWOX5.2-like* will be essential to understand how the broadening of the expression domain results in functional novelty such as determinate growth during rootlet formation.

The comparison of the genome of low P–adapted *L. albus* to *L. angustifolius* revealed that genes related to phosphate use efficiency had expanded in *L. albus* through gene duplications (Xu et al. 2020). One example of such phosphate use efficiency genes is the purple acid phosphatase (PAP) gene family, which is involved in organic P mobilization and expanded in copy number in the *L. albus* genome. Consistently, overexpression of *LaPAP10* in hairy roots increased P content in the root (Xu et al. 2020). Similar investigations into the function of genes/gene families that have expanded in white lupin will be key to unraveling genetic determinants of adaptations to low phosphorus. There is also evidence for shoot-derived sucrose and root auxin signaling as regulators of proteoid root development and function, thus warranting focused analysis of these pathways to uncover the evolution of this adaptive trait (Gilbert et al. 2000; Zhou et al. 2008).

#### Developmental mechanisms for haustoria formation

**Figure 4.**
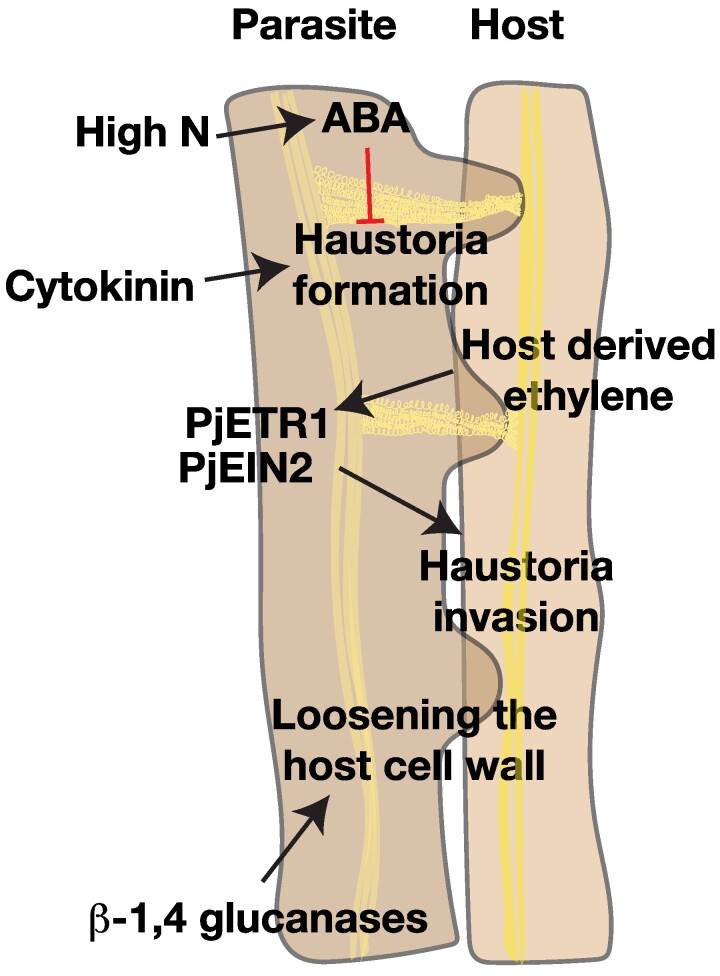
Genetic regulators of parasitic plant-host interactions. The successful invasion of the host by a parasitic plant involves loosening of the cell wall by β-1,4 glucanases and perception of the host-derived ethylene by the parasite. The differentiation and establishment of vascular connections in the haustoria is dependent on cytokinin and ABA signaling.

Parasitic plants sense metabolites, called haustoria-inducing factors, produced by the host to initiate haustoria development. The search for haustoria-inducing factors in *Orobanchaceae* has led to the identification of components of the plant root exudates such as flavonoids, quinones, phenolic acid, and cytokinin. In *S. hermonthica*, the germinating radicle develops into the pre-haustorium involving terminal differentiation of cells in the root. Immediately following germination, the root meristem of *S. hermonthica* is patterned similarly to that of *A. thaliana*. The differentiation into the pre-haustorium involves decrease in *ShPLETHORA1/2* (*ShPLT1/2*) levels, change in PIN polarity, decrease in auxin levels, and increase in cytokinin levels (Xiao et al. 2022). In the facultative parasite *Phtheirospermum japonicum*, lateral haustoria, typically several per root, form from the primary root. A forward genetic screen for regulators of haustoria in *P. japonicum* identified *Pjetr1* (*PjETHYLENERESISTANT1*) and *Pjein2* (*PjETHYLENEINSENSITIVE2*) mutants that showed a delay in termination of haustoria elongation, implicating the role of ethylene in lateral haustoria development (Fig. 4). The haustoria in both *Pjetr1* and *Pjein2* mutants failed to penetrate the host, suggesting that ethylene plays a key role in several stages of parasitic plant infection (Cui et al. 2020). Thus, the perception of host-generated ethylene in the parasite was found to be essential for haustoria invasion. When the haustoria encounters the host, division ceases and differentiation is triggered, allowing invasion and connection to the host vasculature. The parasite uses ethylene produced by the host to determine the timing of haustoria differentiation. Similarly, cytokinin production in the parasite is important for successful infection, suggesting that bidirectional movement of signaling molecules determines parasitic plant infectivity. Recently, cell wall loosening by pectin methyl esterase activity in the outer cell layers of the haustoria was found to be critical for host invasion. On the other hand, inhibitors of pectin methyl esterase activity were expressed in the inner tissue layer, potentially to maintain tissue integrity during host penetration (Leso et al. 2023).

In addition to the regulation of pectin composition of the wall, β-1,4 glucanases secreted by the parasite are essential for loosening the host cell wall and efficient bridging of the vasculature between the host and the parasite (Kurotani et al. 2020) (Fig. 4). The environment surrounding the host and parasite exerts its control on successful parasitism too. Nitrogen-rich conditions were identified to efficiently suppress haustoria formation. Interestingly, high nitrogen increased ABA levels and signaling in the parasite and exogenous ABA treatment suppressed haustoria formation, suggesting ABA to be a negative regulator of haustoria development (Kokla et al. 2022) (Fig. 4).

#### Strategies and challenges for trait engineering

While the pathways described above provide several genetic candidates for engineering root traits, successful engineering may be hampered by uncertainties in the transferability of complex traits across species. In addition to this unpredictability, a number of genes identified to modulate relevant traits are either part of a hormone signaling pathway or a key developmental pathway that has broad functions. Thus, successful engineering would require more precise spatial and temporal control of candidate gene expression and a better approach for selecting genes for manipulating traits (Brophy et al. 2022; Lloyd et al. 2022; Guiziou et al. 2023; Ragland et al. 2024). Comparative studies on closely related species of differing stress tolerances or comparisons between more stress-tolerant wild relatives vs their domesticated counterparts could be one way of selecting more promising candidates. In addition to this, characterization of multiple species that have independently evolved a key trait will help identify the gene space that is amenable for engineering a certain trait. While overexpression or loss of function has been used as a strategy for obtaining stress-tolerant plants, engineering specific anatomical traits would require controlling gene expression precisely over developmental time. Advancements in single cell/spatial transcriptomics and extending these techniques to nonmodel species would be critical for identifying promoters that are specific to a certain cell type (Wang et al. 2024). In addition to this, synthetic biology approaches such as the use of genetic logic gate circuits would be essential for generating spatial patterns where suitable promoters cannot be obtained (Brophy et al. 2022). Perhaps what is most challenging is to engineer responsiveness to the environment. The formation of root anatomical modifications such as aerenchyma and changes to root hair morphology occur only under specific conditions, and engineering these traits to occur constitutively in a species might have adverse effects. Overcoming this would require answering basic questions about environmental perception and how this links to gene function and phenotypic plasticity.

#### Conclusions and future perspectives

In agriculture, no single crop is sufficient to support the food, fuel, fiber, and pharmaceutical needs of the global human population. As such, research on a diverse collection of species is needed to generate transferable knowledge that benefits the cultivation of crops and plants that are targets of conservation efforts. Much of the investment in model systems has focused on plant families with an abundance of crop species (Marks et al. 2021). The *Brassicaceae*, *Solanaceae*, and *Poaceae* families, in particular, have seen a rapid expansion of genome sequences being elucidated and plant species studied at the molecular level. Other plant families are ripe for further exploration as well. For example, the *Asteraceae* family contains several plants used in medicine and horticulture and includes an overrepresentation of invasive species (Hodgins et al. 2016). Limited work has been performed on the anatomical innovations and environmental response pathways that exist here and in many other plant families. This is despite the diversity in ecological contexts and growth forms that exist. Important physiological insight can be gained by exploring how root system anatomy varies, whether growth hormone pathways perform similar functions as in other families, and which gene families are associated with ecological niche expansions.

While botanic gardens and natural ecosystems provide important opportunities to access diverse plant species, a molecular understanding of the pathways conferring environmental stress resilience will be facilitated by the ability of experimental scientists to grow organisms under controlled laboratory conditions. The extraordinary impact that Arabidopsis research has had highlights the importance of identifying species that are accessible to the widest population of researchers for cultivation and study (Provart et al. 2016). However, little information is currently available as to which species in the plant kingdom are readily available from germplasm resources and what the growth condition requirements are to go from seed to seed within a reasonable experimental timeframe. Identifying species with interesting attributes will likely be an important first step before investing in the development of transformation protocols and other tools that enable a molecular-genetic approach. Such resources will enable mechanistic, comparative biology that has the broadest potential for positive impact on our global society.

## Dive Curated Terms

The following phenotypic, genotypic, and functional terms are of significance to the work described in this paper:

ARR12 Gramene: AT2G25180
ARR12 Araport: AT2G25180

EGL3 Gramene: AT1G63650
EGL3 Araport: AT1G63650

MYB36 Gramene: AT5G57620
MYB36 Araport: AT5G57620

CYCD6;1 Gramene: AT4G03270
CYCD6;1 Araport: AT4G03270

AXR1 Gramene: AT1G05180
AXR1 Araport: AT1G05180

PIN2 Gramene: At5g57090
PIN2 Araport: At5g57090

root AmiGo: PO:0009005

UBP14 Gramene: AT3G20630
UBP14 Araport: AT3G20630

IAA13 Gramene: Os03g0742900
IAA13 Araport: Os03g0742900

AUX1 Gramene: Os01g0856500
AUX1 Araport: Os01g0856500

aerenchyma AmiGo: PO:0005702
